# Feature Reviews in Medicinal Chemistry

**DOI:** 10.3390/ph18020260

**Published:** 2025-02-14

**Authors:** Mary J. Meegan

**Affiliations:** School of Pharmacy and Pharmaceutical Sciences, Panoz Institute, Trinity College Dublin, D02 PN40 Dublin, Ireland; mmeegan@tcd.ie

This Special Issue of Pharmaceuticals (“Feature reviews in Medicinal Chemistry”) contains a series of reviews covering a broad selection of topics in Medicinal Chemistry, which are chosen to illustrate important recent results in this fast-evolving interdisciplinary research area that aims to improve human health by developing drugs to combat diseases. The collection contains contributions that are focussed on drug discovery and newly approved drugs together with reviews in specific areas of therapeutics. While small-molecule drug discovery is very well established and dominates FDA-approved drugs with more than 50% of the global drugs market [1,2], there is a growing presence of macromolecules, such as oligonucleotide therapeutics (antisense therapy, siRNA, oligonucleotide conjugates, aptamers), in newly approved therapeutics together with antibodies, antibody–drug conjugates, targeted protein degradation strategies (PROTACs), and CAR-T-cell therapies [3,4]. The reviews collected in this Special Issue illustrate the diversity of contemporary research in medicinal chemistry and demonstrate the challenges and opportunities for scientists in this area. Among the notable FDA approvals in 2024 were resmetirom, which is the first treatment for non-alcoholic steatohepatitis (NASH), givinostat, a nonsteroidal treatment for all types of Duchenne muscular dystrophy, inavolisib (a breast cancer treatment that inhibits and degrades the mutant cancer target PI_3_Ka), and a new schizophrenia treatment called cobenfry (a combination of the muscarine agonist xanomeline and the muscarinic antagonist trospium chloride). The next significant developments in medicinal chemistry in the area of small molecules may include the targeting of protein–protein interactions (PPIs), allosteric modulators, protein degraders, undruggable proteins, and small molecules targeting RNA [5]. These projected trends highlight the dynamic and innovative developments in medicinal chemistry, where small molecules play a central role in the design and development of new medicines designed to target unmet needs. The major disease areas of focus in the next 10 years are predicted to be cancer (targeting specific mutations and overcoming resistance), neurodegenerative diseases (disease-modifying treatments, gene and RNA therapies), cardiovascular, and metabolic diseases, while areas of rare and genetic disorders, autoimmune and inflammatory diseases, and mental health disorders continue to be of major interest. Infectious diseases represent a continuous and increasing threat to human health and welfare; future research is required in the areas of antimicrobial resistance, global viral threats, and neglected tropical diseases. These diseases will benefit from innovations in medicinal chemistry, molecular biology, AI, and personalised medicine.

The design of potent, selective PDE5 inhibitors (PDE5-Is) is presented in the review by Abadi et al., which provides a comprehensive overview of the biological function, limitations, and challenges of these molecules (Contribution 1). The therapeutic potentials, relevant clinical trials, future prospects, and emerging uses are discussed, which are of significant relevance to current and future research in the topic of PDE5-Is in both academia and industry areas [6]. Phosphodiesterase 5 (PDE5) inhibitors have an important role in the nitric oxide/cGMP pathway and modulate many biochemical and pathological processes. They are successfully used in the clinical treatment of male erectile dysfunction (ED) and pulmonary arterial hypertension (PAH). An updated assessment of the potential applications of PDE5-Is in the treatment of cognitive functions, heart failure, multiple-drug resistance in cancer therapy, immune diseases, and systemic sclerosis is presented. PDE5-Is demonstrating multi-targeted activity are discussed together with the recently reported PDE5 allosteric inhibitors, which demonstrate significant isoenzyme selectivity.

A review of the synthesis and bioactivity of bisindolyl maleimides and indolylmaleimide derivatives is presented by McCarthy et al. (Contribution 2). Bisindolylmaleimide (BIM)-type heterocyclic compounds are found in natural sources such as arcyriarubin A, a potent protein kinase C (PKC) and PKA inhibitor and also a potent selective inhibitor of human HCMV replication; they are biosynthetically related and act as immediate synthetic precursors of indolocarbazoles [7]. In this review, new synthetic approaches to BIM-type compounds are presented, together with their associated bioactivities. Potential clinical applications are discussed. Synthetic studies relevant to this class of compounds are diverse, and there have been many recent developments of both potent and selective protein kinase inhibitors based on the bisindolylmaleimide scaffold structure. Clinical BIM protein kinase inhibitors include ruboxistaurin (an investigational drug for diabetic retinopathy) and enzastaurin, (intended for the treatment of solid and haematological cancers) developed as an isozyme-specific inhibitor of protein kinase Cβ (PKCβ), which is involved in both the AKT and MAPK signalling pathways relevant in many cancers. Additional bioactivities reported for BIMs include the inhibition of calcium signalling and antimicrobial activity. The BIM molecular scaffold is useful for chemical elaboration, as it can be functionalised and manipulated using appropriate chemical transformation, allowing the synthesis of new derivatives with interesting biological profiles. In this review, the newer synthetic approaches to BIM-type heterocycles are presented, together with their unique biological activities and potential clinical applications.

The topic of epilepsy and its treatment is presented in the review by Malaník et al., which poses the question “Treating Epilepsy with Natural Products: Nonsense or Possibility?” (Contribution 3). Epilepsy is a neurological disease condition that affects the brain and causes frequent seizures. The underlying mechanism of an epileptic seizure is excessive and abnormal neuronal activity in the cortex of the brain. Although effective antiepileptic drugs are available for patients, there is an urgent need for accessible and cost-effective anti-seizure medication for the condition [8]. The most commonly prescribed anti-seizure drugs are carbamazepine, phenytoin, gabapentin, pregabalin, levetiracetam, clonazepam, phenobarbital, tiagabine, valproate, and topiramate; however, many of these drugs have side-effects. Studies available to date suggest that natural products show mechanisms of action similar to those of clinically used drugs. The molecular targets of a number of natural plant products showing anticonvulsant activity are summarised, allowing the most promising therapeutic candidates to be selected, e.g., huperzine A, thymoquinone, and quinidine. Natural products are also suitable templates for the design of semisynthetic derivatives with improved efficacy and pharmacokinetic properties. A number of natural products have demonstrated anticonvulsant activity in in vivo models of epilepsy and with clinical data. The authors indicate that these natural plant-derived products may have potential as anti-seizure medications. Epidiolex^®^ (cannabidiol) is approved for the treatment of some rare epilepsies. This review is presented as an introduction to the topic and will serve as a basis for future research into plant derived products, which could be used for epilepsy and related neurological disorders.

Metalloenzymes are a broad group of enzymes that use a heavy metal cation coordinated into their active centre as a cofactor in the enzyme active site. The enzymes promote a diverse range of biological reactions including hydrolysis reactions and oxidation/reduction processes. Metalloenzymes have an important role in the regulation of many biological functions [9]. Metalloenzymes are central to the regulation of a wide range of essential viral and parasitic functions, including protein degradation, nucleic acid modification, and many others. The review of Zoidis et al. shows the recent advances that have been made in targeting the metalloenzymes of viruses and parasites and presents the latest advancements in targeting metalloenzymes that play a crucial role in the pathogenesis of specific diseases (Contribution 4). Approximately one-third of all proteins are believed to be metalloproteins, and some of them are responsible for crucial viral and parasitic functions. Infectious diseases have a significant impact on human health globally; therefore, the design and development of inhibitors of metalloenzymes is an important approach to advancing therapeutics for infectious diseases. Metal-chelating agents have been studied as antivirals and antiparasitic agents, resulting in the discovery of important classes of metal-dependent enzyme inhibitors. In this review, the recent research in targeting the metalloenzymes of viruses and parasites, which have been identified as important targets in global public health, are discussed. Specific targets identified include influenza virus RNA-dependent RNA polymerase, hepatitis B virus (HBV) polymerase, hepatitis C virus nonstructural protein 5B, *Trypanosoma brucei* 6-oxopurine phosphoribosyltransferase, *Trypanosoma cruzi* carbonic anhydrase, and human immunodeficiency virus (HIV) integrase and ribonuclease. The metal-chelating inhibitors of viral and parasitic metalloproteins are identified as very promising and safe therapeutic agents for the treatment of serious viral and parasitic diseases.

The outbreak of severe acute respiratory syndrome coronavirus 2 (SARS-CoV-2) in 2019 resulted in the global coronavirus disease 2019 (COVID-19) pandemic. The COVID-19 vaccines have reduced the spread of COVID-19 and reduced the severity and death caused by COVID-19. To date, no small molecule or peptidomimetic therapy has been found to selectively target the virus, and there is an urgent need for new clinical therapies. Rozas et al. present an overview of two proteases of different origins (viral and human host) that play an important role in the infection by SARS-CoV-2: the main protease of SARS-CoV-2 (M^Pro^) and the host transmembrane protease serine 2 (TMPRSS2), (Contribution 5). The replication cycle of SARS-COV-2 is summarised to indicate the distinct roles of MPro and TMPRSS2 in the viral replication cycle [10]. These two proteases have become important targets for the development of antiviral agents to treat COVID-19. The characteristic features of the agents currently approved to treat COVID-19 [remdesivir, molnupiravir, ensitrelvir and paxlovid (i.e., nirmatrelvir and ritonavir)] are summarised together with a discussion of some of the most recently reported inhibitors of the viral M^Pro^ [simnotrelvir, myricetin, ebselen and lycorine] and the host TMPRSS2, such as otamixaban. The mechanism of action of each protease is presented, together with a summary of the most recent computational approaches used by researchers to design novel M^Pro^ and TMPRSS2 inhibitors. Some approaches to the design of dual-action inhibitors are discussed, which simultaneously inhibit the viral proteases M^Pro^ and PL^Pro^, the viral protease M^Pro^, the spike S protein, and viral M^Pro^ and host TMPRSS2 proteases.

Gliomas comprise about 30 percent of all brain tumours and central nervous system tumours and 80 percent of all malignant brain tumours. Gliomas are the most common primary intracranial neoplasms in adults and are a leading cause of cancer-related morbidity and mortality in the United States. Glioblastoma (GBM) is extremely difficult to treat, and most treatment options for patients are ineffective and mainly palliative, despite advances in targeted therapeutics [11]. Pfeffer et al. present a review of the STAT3-regulated autophagy pathway in GBM and describe recent research findings that suggest GBM tumours are more sensitive to the excessive overactivation of autophagy leading to autophagy-dependent cell death (Contribution 6). GBM cancer stem cells (GSCs) play critical roles in tumour formation and progression, metastasis, and relapse; they are resistant to most therapeutic strategies as they can adapt to a tumour microenvironment of hypoxia, acidosis, and lack of nutrients. The role played by autophagy in GBM pathogenesis is complex. Autophagy may promote and maintain the stem-like state of GSCs as well as their resistance to cancer treatment; however, autophagy may also exhibit anti-tumour effects, which may be related to the role of the STAT3 transcription factor in autophagy. Autophagy regulation is a possible route to overcome the therapeutic resistance of GBM, while autophagy modulation may specifically target the highly therapy-resistant GSC population. Future studies are required to identify the potential roles that regulation of autophagy in GBM can play, potentially inhibiting GBM tumorigenesis and overcoming the therapeutic resistance of GBM to chemotherapy and radiotherapy.

A comprehensive review of the synthetic methodologies and therapeutic potential of indole-3-carbinol (I3C) and its derivatives is presented by Duranti, Pandolfi et al. (Contribution 7). Indole-3-carbinol (I3C) is a secondary metabolite contained in vegetables belonging to the *Brassicaceae* family and is of particular interest for its biological and pharmacological properties [12]. I3C, together with its major metabolite 3′-diindoylmethane (3,3′-DIM or DIM), and others have important biological properties including the prevention and treatment of inflammatory origin diseases. In this review the synthetic procedures through which I3C and its main exogenous derivatives have been obtained are summarised. The biosynthesis processes for I3C endogenous derivatives are discussed, together with the main biosynthetic oligomer compounds derived from I3C. The important characteristic biological properties obtained from bioassays and in vivo pharmacological tests of these compounds are presented, including I3C antitumour, anti-inflammatory, antiviral, antimicrobial, antioxidant, antiobesity, and neuroprotective activity. In vitro and in vivo activity of I3C derivatives is summarised while the mechanisms of action of the most important compounds are considered. The potential social impact that the molecules may have in the prevention and treatment of respiratory tract disorders and cancer is summarised. The evaluation of the natural dimeric, trimeric, or tetrameric derivatives of I3C are discussed, which contribute to the understanding of the structural requirements for the activity of these potentially useful compounds based on the natural product Indole-3-carbinol and its derivatives.

The role of cyclooxygenase-2 (COX-2) as a target of anticancer agents is reviewed by Ahmad, Zaki et al. (Contribution 8). Several novel synthesised scaffolds having dual anticancer and COX-2 inhibitory applications have been reported. While chemotherapy is a clinically beneficial treatment for many cancer patients, drug resistance and severe toxic side-effects are a significant disadvantage of many anticancer therapies. Cyclooxygenase-2 (COX-2) is frequently over-expressed in many types of cancers; it plays a role in cancer development and progression and also in cancer cell resistance to chemotherapy and radiotherapy. COX-2 is identified as a significant target for the synthesis of new anticancer agents [13]. This review provides a summary of recently reported heterocyclic and hybrid anticancer molecules having dual anticancer and COX-2 inhibitory properties together with analysis of the key ligand–protein interactions with the COX-2 enzyme active site. Exploration of these diverse molecular structures offers potential for the optimisation of novel selective lead compounds having dual anticancer and COX-2 activity.

Fungal infections are a serious global concern with evidence of increasing hospitalisation and mortality rates. There is a need for novel antifungal agents due to rise in resistance to the clinically available drugs such as the azoles [14]. In this comprehensive review of new antifungal agents with azole moieties, Sousa et al. present current developments in the chemistry and biology of new azole agents, including those in clinical trials, together with complementary assays, e.g., toxicity and susceptibility assays and molecular docking studies (Contribution 9). Additional innovative molecules with promising antifungal activity are also discussed together with relevant structure–activity relationship (SAR) studies. The azole moiety is recognised as a privileged structure, with compounds having distinct pharmacophores and SAR. The introduction of azoles such as 1,2,3-triazole in bioactive natural product conjugates resulted in the identification of compounds with significative activity as antifungal agents, including against resistant strains.

Chalcones are considered as privileged scaffolds in medicinal chemistry due to the presence of the α,β-unsaturated ketone functionality [15]. The α,β-unsaturated ketone offers an electrophilic system as a potential target for enzymatic action of the compounds. Covalent interactions of chalcones with biological targets occur via the Michael acceptor activity of the α,β-unsaturated carbonyl system, while radical scavenging or reduction may also occur. Many chemical modifications of the chalcone structure have been reported, which influence their pharmacological activity. In the present review, “Chalcone: A Promising Bioactive Scaffold in Medicinal Chemistry”, Ramani et al. summarise the chalcone structures isolated from natural sources and illustrate the important synthetic methods for these compounds (Contribution 10). The diverse biological activities demonstrated by chalcones against infectious and non-infectious diseases are discussed, together with their characteristic structure–activity relationships. This comprehensive review provides useful information for medicinal chemists to guide development of candidate lead compounds with a variety of potential medical applications.

Paprocka et al. present a comprehensive review of the structure and biological activities of amidrazone derivatives reported in the years 2010–2022 (Contribution 11). Amidrazones are of general interest as they are extensively used in synthesis, industry, and agriculture, and many amidrazone derivative possess significant biological activities. The structures described in this review include amidrazone derivatives, aminoguanidine derivatives, complexes obtained using amidrazones as ligands, and some cyclic compounds obtained from amidrazones. The relevant biological activities of interest include tuberculostatic, antibacterial, antifungal, antiparasitic, antiviral, anti-inflammatory, cytoprotective, and antitumour compounds [16]. The associated mechanisms of action and toxicity of the selected amidrazone derivatives are discussed. Amidrazone compounds are identified with potential for progression to preclinical or clinical research, while the identification of older amidrazone drugs with new therapeutic targets (repositioning) indicate that amidrazone derivatives may be a potential source of new therapeutic substances.

The reviews presented in this Special Issue of Pharmaceuticals “Feature reviews in Medicinal Chemistry” contain contributions that are focussed on diverse areas of contemporary medicinal chemistry. These reviews highlight both the challenges and opportunities that are encountered in the discovery and development of both novel small-molecule and biological drugs. The applications of innovative research in medicinal chemistry are outlined, and the direction and potential for future research in these areas are discussed.

## Data Availability

Data are contained in the article.
